# Identification and Comparative Analysis of CBS Domain-Containing Proteins in Soybean (*Glycine max*) and the Primary Function of *GmCBS21* in Enhanced Tolerance to Low Nitrogen Stress

**DOI:** 10.3390/ijms17050620

**Published:** 2016-04-26

**Authors:** Qingnan Hao, Weijuan Shang, Chanjuan Zhang, Haifeng Chen, Limiao Chen, Songli Yuan, Shuilian Chen, Xiaojuan Zhang, Xinan Zhou

**Affiliations:** 1 Institute of Oil Crops Research, Chinese Academy of Agriculture Sciences, Wuhan 430062, China; haoqingnan@126.com (Q.H.); shang-weijuan2006@163.com (W.S.); zhangchanjuan@aliyun.com (C.Z.); smartchf@163.com (H.C.); ccllmm_008@163.com (L.C.); yyyyy-0909@163.com (S.Y.); 2 Key Laboratory for Biological Sciences of Oil Crops, Chinese Academy of Agricultural Sciences, Ministry of Agriculture, Wuhan 430062, China; chenshuilianjy@163.com (S.C.); xjzhang@oilcrops.cn (X.Z.)

**Keywords:** CBS domain, soybean, function analysis, low nitrogen stress tolerance

## Abstract

Nitrogen is an important macronutrient required for plant growth, and is a limiting factor for crop productivity. Improving the nitrogen use efficiency (NUE) is therefore crucial. At present, the NUE mechanism is unclear and information on the genes associated with NUE in soybeans is lacking. cystathionine beta synthase (CBS) domain-containing proteins (CDCPs) may be implicated in abiotic stress tolerance in plants. We identified and classified a CBS domain–containing protein superfamily in soybean. A candidate gene for NUE, *GmCBS21*, was identified. *GmCBS21* gene characteristics, the temporal expression pattern of the *GmCBS21* gene, and the phenotype of *GmCBS21* overexpression in transgenic *Arabidopsis thaliana* under low nitrogen stress were analyzed. The phenotypes suggested that the transgenic Arabidopsis thaliana seedlings performed better under the nitrogen-deficient condition. *GmCBS21*-overexpressing transgenic plants exhibit higher low nitrogen stress tolerance than WT plants, and this suggests its role in low nitrogen stress tolerance in plants. We conclude that *GmCBS21* may serve as an excellent candidate for breeding crops with enhanced NUE and better yield.

## 1. Introduction

Nitrogen (N) is one of the main nutrient elements for plant growth and development and thus plays a very important role in plant productivity and crop yield [1]. An appropriate increase in the amount of nitrogenous fertilizer is one of the measures necessary to obtain a high crop yield. However, excessive application of nitrogen fertilizer not only reduces crop nitrogen uptake and utilization efficiency, but also results in reduced crop production efficiency, a waste of resources, environmental pollution and other issues [2,3]. To increase crop yield as a major goal, studies on how to reduce the amount of nitrogen fertilizer and increase crop nitrogen utilization efficiency are very important for the sustainable development of agriculture.

The search to identify genes that improve the nitrogen use efficiency (NUE) of crop plants is ongoing. In our previous study, two soybean genotypes were grown under N-limited conditions; a low N-tolerant variety (Pohuang) and a low N-sensitive variety (84–70) were used for transcriptome analysis. A number of soybean genes were differentially expressed between the two varieties under N-limited conditions. Some of these genes may be candidates for improving NUE [4]. One of the genes, *GmCBS21*, was cloned and analyzed.

The *GmCBS21* gene encoded a protein containing the cystathionine beta synthase (CBS) and DUF21 domains [5]. DUF21 domains are transmembrane regions with unknown function adjacent to CBS domains. CBS domains are also widely present in other proteins such as inosine-5′-monophosphate dehydrogenase (IMPDH) [6], voltage-gated chloride channels (CLC) [7] and AMP-activated protein kinase (AMPK) [8]. CBS domains regulate the activity of associated enzymatic and transporter domains in response to binding molecules with adenosyl groups such as AMP and ATP, or *S*-adenosylmethionine [9]. CBS domains are often found in proteins that contain other domains having enzymatic, membrane transporter or DNA-binding activities. However, proteins that contain only CBS domains are also found frequently, particularly in prokaryotes. These standalone CBS domain proteins might form complexes upon binding to other proteins (such as kinases) for interacting with and regulating. The CBS domain-containing proteins (CDCPs) are composed of a superfamily of proteins conserved during evolution. The main feature of these proteins is that they are usually connected to two or four CBS domains [10].

Although the CBS function is unclear, mutations in some human CDCPs lead to genetic diseases [11]. Studies on CDCPs in plants were recently initiated. Some studies revealed that CBSX (protein with only one pair of CBS domains without any other protein domains) proteins directly regulate the activation of thioredoxins and thereby control cellular H_2_O_2_ levels and modulate both plant development and growth [12]. Functional analysis showed that mature CbCBS (CBS domain–containing protein from *Coleus blumei*) may act as a sensor of cellular energy status and may directly or indirectly regulate cellular energy levels to increase the ATP content in mitochondria during periods of metabolic stress in senescent leaves [13]. Some studies found that many of the CDCPs with unknown function in *Arabidopsis* and *Oryza* were related to abiotic stress [10]. Singh *et al.* found that *OsCBSX4* was regulated by stress and development, and overexpression of *OsCBSX4* in model tobacco (*Nicotiana tabacum*) plants resulted in enhanced tolerance against various abiotic stresses such as salinity, heavy metals, and oxidative stress [14]. *OsBi1* is a CBS-containing gene that is implicated in the resistance of rice plants to brown plant hoppers and responds to biotic and abiotic stress [15].

In this study, we identified and carried out a comparative analysis of CBS domain–containing proteins in soybean. Furthermore, one of the genes, *GmCBS21*, was cloned and its implication in low nitrogen stress tolerance was analyzed. *GmCBS21* overexpression in *Arabidopsis thaliana* plants induced higher tolerance to low nitrogen conditions, suggesting a role in nitrogen stress responses.

## 2. Results

### 2.1. Identification, Characterization and Classification of CBS Domain-Containing Proteins in Soybean

Based on the domain sequences of the CBS superfamily members of *Arabidopsis* and rice, we searched the CBS genes in the soybean database of NCBI by BLASTP, and searched in the phytozome soybean genome database using the keyword (CBS domain). A total of 71 putative CBS genes were identified by removing redundant sequences and different transcripts of the same gene. All these putative CBS protein sequences were predicted to include one or two CBS domains. The 71 soybean CBS genes were named *GmCBS1* to GmCBS71 according to their chromosomal positions. Detailed information about soybean CBS genes is given in Table S1. The gene-encoded peptides’ length ranged from 166 (Glyma.16G086600) to 801 (Glyma.11G004600) amino acids, *M*w from 18.6 to 88.2 kDa and pI from 4.90 to 9.30. Subcellular localization prediction indicated that 24 genes were localized in the plasma membrane, 21 genes were localized in the chloroplast, 19 genes were localized in the cytoplasmic, four genes were localized in the nuclear, two genes was localized in the mitochondrial, and one gene was localized in the endoplasmic reticulum.

In previous studies of *Arabidopsis* and rice, all the CDCPs were divided into two groups: one CBS domain-containing proteins and two CBS domain-containing proteins. One CBS domain-containing proteins were further classified into seven subgroups based on the additional domain(s) present in their sequences [10]. Of the 71 soybean CBS identified genes, 10 presented a single CBS domain whereas 15 contained two CBS domains. All the other members of the group presented additional domains different to the CBS domain. Thus, three genes contained a domain of unknown function (DUF21) (PF01595) and a CorC_HlyC domain (PF03471) at the N-terminus and C-terminus of the proteins, respectively, whereas 12 members contained the voltage-gated chloride channel domain (or motif) at the N-terminus and two genes the pentatricopeptide repeat (PPR) motif. Finally seven genes, two genes and one gene presented *Phox/Bemp1* (PB1), inosine-5′-monophosphate dehydrogenase (IMPDH) (PF00478) or the sugar isomerase (SIS) domains, respectively.

### 2.2. Phylogenetic Motif Recognition, Genome Distribution and Gene Structure of the CBS Superfamily Genes in Soybean

**Figure 1:**
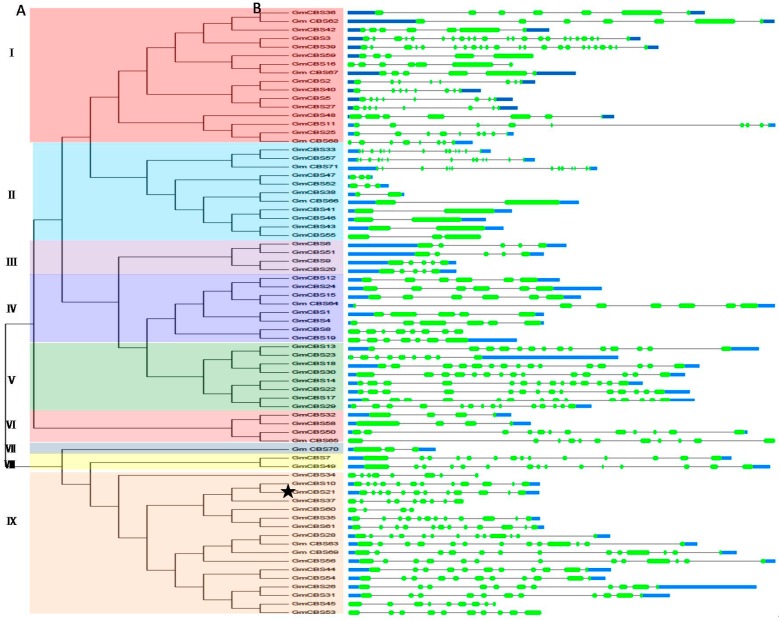
Phylogenetic analysis and structure of soybean CBS genes. (**A**) Phylogenetic tree construction of soybean CBS genes based on the full-length deduced amino acid sequences using MEGA 5.0 (Koichiro Tamura, Daniel Peterson, Nicholas Peterson, Glen Stecher, Masatoshi Nei, and Sudhir Kumar) by the maximum likelihood method with 500 bootstrap replicates. The tree showed nine major phylogenetic groups (group I to IX) indicated by different colored background. The star marked our candidate gene *GmCBS21*; (**B**) Exon/intron structures of *GmCBS* genes. Green boxes represent exons, black lines indicate introns, and blue boxes represent untranslated region (UTR).

To determine the phylogenetic relationships among the different members of the CBS superfamily in soybean, phylogenetic analysis based on alignments of the 71 full-length CBS protein sequences was performed. The phylogenetic analysis indicated that 71 CBS superfamily members can be divided into nine groups (Figure 1A). The structure of the corresponding genes was shown in Figure 1B.

The members within each group showed similar exon/intron structures. Group I comprised 16 members, and most of them are voltage-gated chloride channel proteins except *GmCBS2*, *GmCBS40*, *GmCBS5* and *GmCBS27*. Group II was formed by 11 members which are all AMP-activated protein kinases. Group III was formed by four members with five exons. Group IV was constituted by eight members, which may be AMP-activated protein kinase. Group V was formed by eight members with 14 exons, except *GmCBS23*, which has nine exons. Group VI was formed by four members. Group VII was constituted by one member with three exons, which contain a SIS domain. Group VIII was formed by two members with 14 and 15 exons. Group IX was formed by 17 members which are all proteins of unknown function. Several pairs of GmCBS proteins have a high degree of homology in the terminal nodes, and they are putative paralogous pairs. A total of 30 putative paralogous pairs were identified, with sequence identity ranging from 65% to 99% (Table S2).

**Figure 2:**
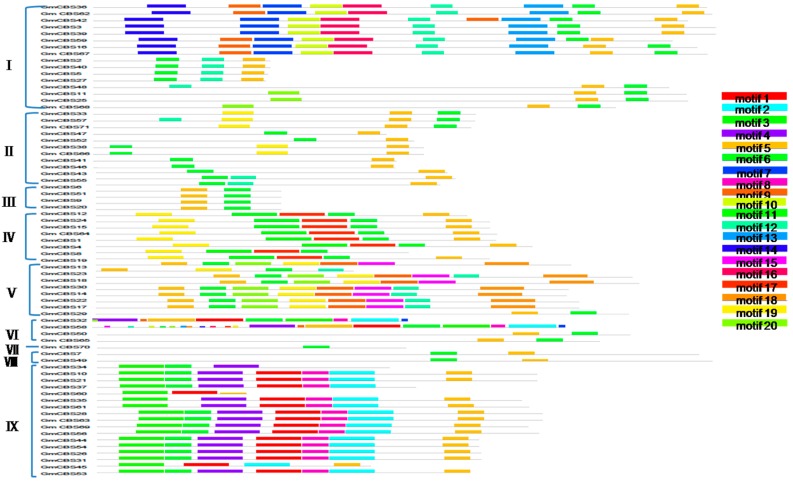
Schematic distribution of the conserved motifs in soybean CBS superfamily by MEME. The conserved motifs were identified in the proteins of every group. Each colored box below the tree represents the conserved motifs. Motif 2, 5, 6, 8, 9, 15 and 19 were CBS-related motifs.

To further reveal the diversification of CBS genes in soybean, putative conserved motifs were predicted by the program MEME. A total of 20 distinct motifs were identified in all 71 CBS domain-containing proteins. The schematic distribution of the 20 motifs among the different gene groups is shown in Figure 2, and the identified multilevel consensus sequence for the motifs is shown in Table S3. Motif 1, motif 2 and motif 8 mainly existed in group VI and group IX, and were TlyC CBS_pair_CorC_HlyC. Motif 3 and motif 4 mainly existed in group VI and group IX, and had the DUF domain. Motif 5 was present in most of the GmCBS domains (except GmCBS70 and GmCBS37), and was CBS_pair_29. Motif 6 was present in most of the GmCBS domains (except GmCBS35, GmCBS45, GmCBS60 and GmCBS61), and was CBS_pair_10. Motif 7, motif 10, motif 13, motif 14 and motif 16 were present in group I, and were Voltage_gated_ClC. Motif 9 was CBS_pair_CAP-ED_DUF294_PBI_assoc, and was present in the group I, group V and group VI. Motif 15 was present in group V, and was CBS_pair_CAP-ED_DUF294_PBI_assoc. Motif 18 was present in group V, and was PB1. Motif 18 was present in group II, group IV and group V, and was CBS_pair_10.

**Figure 3:**
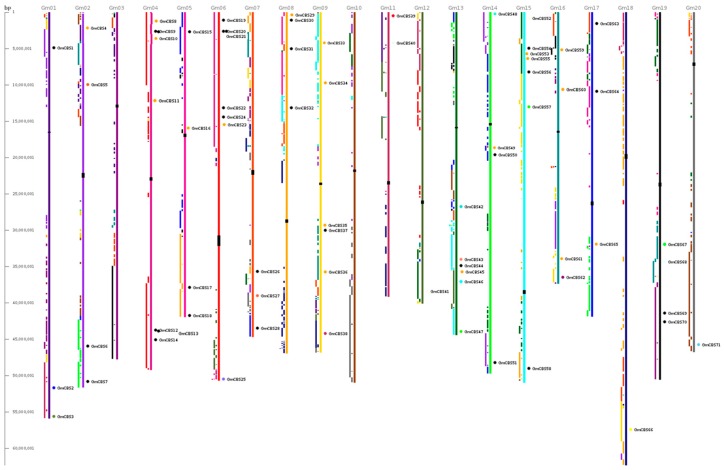
Chromosomal location and region duplication of GmCBS genes. The schematic diagram of genome-wide chromosome organization and segmental duplication was made from the CViT genome search and synteny viewer at the Legume Information System. Colored blocks to the left of each chromosome show duplications with chromosomes of the same color.

The physical locations of the CBS genes on soybean chromosomes are shown in Figure 3. Seventy-one soybean CBS genes were unevenly distributed on 18 chromosomes, except for chromosomes 3 and 10. Among these chromosomes, chromosome 6 and chromosome 15 had the largest number of CBS genes with seven, followed by chromosomes 4, 9, 13 with six. However, chromosomes 12, 18, 20 only had one CBS gene.

### 2.3. The Expression Pattern and Subcellular Localization of GmCBS21

**Figure 4:**
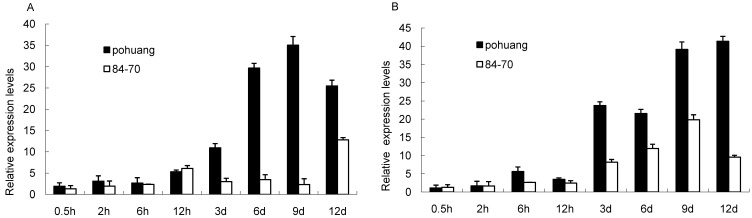
The *GmCBS21* transcript is induced by low nitrogen stress: (**A**) The expression in leaf and stem; (**B**) The expression in root. Three independent experiments were performed, and values are shown as means and error bars indicate standard deviation (*n* = 3).

**Figure 5:**
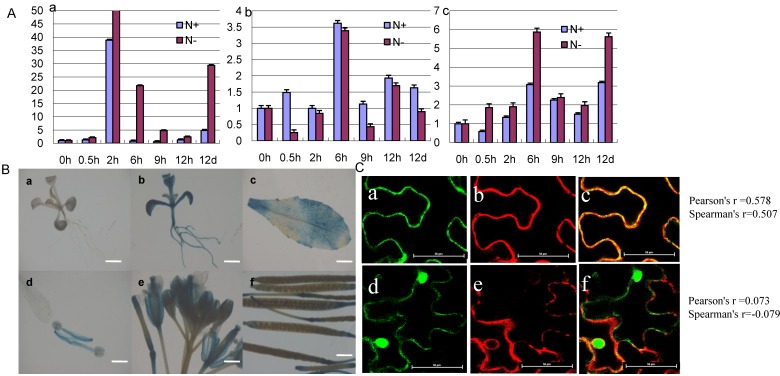
Expression patterns of *GmCBS21* and subcellular localization of *GmCBS21*. (**A**) Relative expression profiles of soybean *GmCBS21* in various organs. Soybean seedlings (Pohuang) were treated with normal nitrogen (N+) and low nitrogen (N−); samples were taken at different time (after treatment 0, 0.5, 2, 6, 9, 12 h, 12 days). (**a**) Relative expression profiles of soybean *GmCBS21* in leaves; (**b**) Relative expression profiles of soybean *GmCBS21* in stems; (**c**) Relative expression profiles of soybean *GmCBS21* in roots. N+ was basic Hoagland’s nutrient solution. N− was basic Hoagland’s nutrient solution with 1/10 N concentration. Three independent experiments were performed, and values are shown as means and error bars indicate standard deviation (*n* = 3); (**B**) Expression of the GmCBS21:GUS reporter gene in transgenic *Arabidopsis* plants. (**a**) Expression of the GmCBS21:GUS reporter gene in seedlings of wild type; (**b**) Expression of the GmCBS21:GUS reporter gene in transgenic seedlings; (**c**) Expression of the GmCBS21:GUS reporter gene in transgenic leaf; (**d**) Expression of the GmCBS21:GUS reporter gene in transgenic stamens; (**e**) Expression of the GmCBS21:GUS reporter gene in transgenic flower; (**f**) Expression of the GmCBS21:GUS reporter gene in transgenic siliques; Scale bars: 1 cm (**a**–**c**); 1.5 cm (**d**,**e**); 0.5 cm (**f**); (**C**) Subcellular localization of the GmCBS21 protein. Subcellular localization of the fused pJG053-35s::GmCBS21 -GFP in tobacco leaves cells. The pJG053::GFP construct was used as the control. (**a**) Green Fluorescent Protein (GFP )channel showing GmCBS21::GFP expression; (**b**) Red Fluorescent Protein (RFP) channel showing pm-rk CD3 mCherry marker expression; (**c**) co-localization of GmCBS21::GFP with the pm-rk CD3-1007 mCherry marker; (**d**) GFP channel showing pJG053::GFP expression; (**e**) RFP channel showing pm-rk CD3 mCherry marker expression; (**f**) co-localization of pJG053::GFP with the pm-rk CD3-1007 mCherry marker. Statistical analysis of the co-localization was done using Pearson’s (rP) and Spearman’s (rS) correlation factors [16]. Bar = 50 μm.

In our former Digital Gene Expression (DGE) data libraries (N-tolerant variety (Pohuang) *vs.* low N-sensitive variety (84–70)), *GmCBS21* was a differentially expressed gene under N-limited conditions (Table S4). qRT-PCR analysis reconfirmed the low nitrogen stress–induced upregulation in soybean, with a higher relative expression in Pohuang compared with 84–70 (Figure 4). The function of a protein is tightly correlated with its expression pattern and subcellular localization. First, using qRT-PCR, we investigated the expression level of *GmCBS21* in different organs of Pohuang. qRT-PCR showed that the *GmCBS21* gene was expressed in the leaves, stems, and roots (Figure 5A). The *GmCBS21* gene was significantly upregulated after low N treatment in leaves and roots, but was downregulated in stems. The expression of *GmCBS21* was significantly upregulated in leaves and roots at 6 h and 12 days. In stems, the expression of *GmCBS21* was significantly downregulated at 0.5, 9 h, and 12 days (Figure 5A). These results indicate that *GmCBS21* may be involved in the response to low N stress.

In order to examine the tissue expression patterns of *GmCBS21*, we performed histochemical expression analysis of the gene using transgenic *Arabidopsis* plants carrying promoter-GUS fusion report systems. T3 homozygous transgenic plants were used for the GUS assays. The results showed that GUS activity was detected in seedlings and all tissues and organs examined, including the leaf, root, stamens, flower, and siliques (Figure 5B).

To determine the cellular localization of the GmCBS21 protein, we introduced the pJG053-35s: GmCBS21-GFP and pJG053-GFP constructs into tobacco leaves cells using *Agrobacterium*-mediated transformation. Green fluorescence of pJG053-35s:GmCBS21-GFP occurred mainly in the plasma membrane, whereas that of pJG053-GFP alone was distributed throughout the cell (Figure 5C).

### 2.4. Prokaryotic Expression and Cystathionine β-Synthase Activity Assay

In order to measure the cystathionine β-synthase of *GmCBS21* *in vitro*, we expressed and purified the protein using a bacterial expression system. The protein was expressed in soluble form with a higher protein activity. The purified proteins were analyzed using SDS-PAGE (Figure S1).

The purified proteins were used to determine cystathionine β-synthase activity. The results are shown in Table S5. The assay group absorbance value and the control group absorbance value were similar. The specific activity of purified cystathionine β-synthase was 0.37 U/mg (specific activity). Based on this value, it can be considered that the protein does not have cystathionine β-synthase activity.

### 2.5. Phenotype of GmCBS21 Overexpression Transgenic Seedlings under Low Nitrogen Stress

A *GmCBS21* overexpression vector was constructed (Figure S2A) and used to transform *Arabidopsis*, resulting in *GmCBS21*-OXP seedlings. RT-PCR showed that *GmCBS21* was constitutively expressed in all 11 T2 *Arabidopsis* lines (Figure S2B). These findings indicated that all 11 lines would be useful in the analysis of stress tolerance. L1, L3, and L8 were chosen for further analysis.

**Figure 6:**
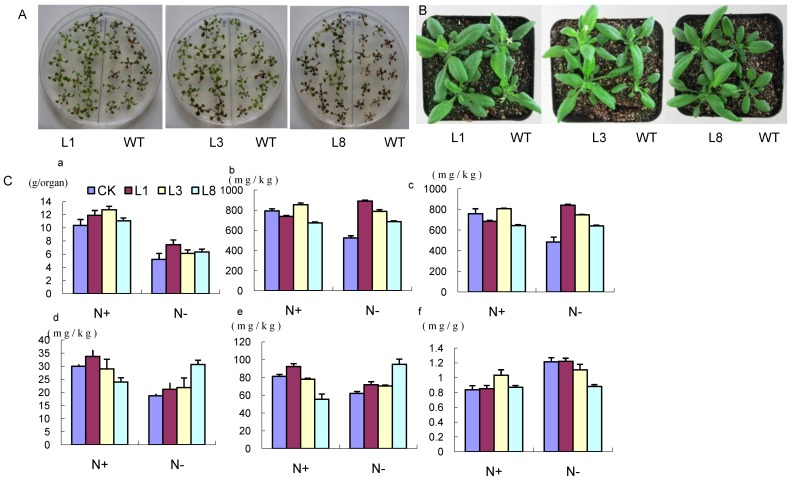
Enhanced low nitrogen stress tolerance in plants overexpressing *GmCBS21*. (**A**) The phenotype of transgenic plants and wild-type plants grown in low nitrogen medium. L1, L3 and L8 were transgenic lines. WT was wild-type control line; (**B**) The phenotype of transgenic plants and wild-type plants grown in low nitrogen vermiculite; (**C**) Several physiological indexes of transgenic plants and wild-type plants grown in low N and normal N condition. L1, L3 and L8 were transgenic lines. CK was wild-type control line. (**a**) Fresh weight of wild-type and *GmCBS21* plants; (**b**) Total nitrogen concentration of wild-type and GmCBS21 plants; (**c**) Nitrate-N concentration of wild-type and *GmCBS21* plants; (**d**) Ammonium nitrogen concentration of wild-type and *GmCBS21* plants; (**e**) Non-protein nitrogen concentration of wild-type and GmCBS21 plants; (**f**) Soluble sugar contents of wild-type and *GmCBS21* plants. L1, L3 and L8 were transgenic lines. WT was wild-type control line. *Arabidopsis thaliana* seedlings were treated with normal nitrogen (N+) and low nitrogen (N−). Seven-day-old plants grown on 1/2 MS medium were transferred to vermiculite supplied with 1/2 Hoagland’s solution and left to bolt (two weeks). Then, they were transferred to normal 1/2 Hoagland’s solution and low nitrogen 1/2 Hoagland’s solution, respectively. After nine days in the low nitrogen solution, all plants were harvested. Data represent as the mean value ± S.E. of three replicates.

Low nitrogen tolerance was evaluated in both seedlings and adult plants. In nitrogen-deficient medium, non-transgenic (NT) and transgenic lines (TL) seedlings exhibited the typical nitrogen-deficient phenotype consisting in discoloration of leaves and less vigorous growth. However, this deficient phenotype was clearly much reduced in TL plants (Figure 6A). In adult plants, all three transgenic lines had significantly larger rosettes than the wild type after nine days of treatment in low nitrogen solution (Figure 6B). The phenotypes suggested that TL performed better under the nitrogen-deficient condition.

### 2.6. Physiological Indexes of GmCBS21 Overexpression Transgenic Seedlings under Low Nitrogen Stress

The fresh weight, total nitrogen, ammonium nitrogen, nitrate nitrogen, and non-protein nitrogen concentrations were measured in NT and TL plants. First, we compared the physiological indexes of soil-grown NT and *GmCBS21* overexpression plants under a normal nitrogen condition (HN). Results showed that the fresh weight of TL was higher than NT, with a maximum 1.23-fold increase in L3 relative to the NT (Figure 6C, panel a). The results of total nitrogen, ammonium nitrogen, nitrate nitrogen, and non-protein nitrogen concentrations are shown in Figure 6C, panels b–e N+. There was no significant difference between the NT and TL plants. After nine days of treatment in low nitrogen solution, the fresh weight was lower than that measured, and TL had a higher weight than the NT, with a maximum 1.43-fold increase in L1. The concentrations of all types of nitrogen were higher in TL than NT. Specifically, the total nitrogen and nitrate nitrogen concentrations were significantly higher, *i.e.*, 1.31- to 1.70-fold and 1.32- to 1.74-fold higher, respectively, in the transgenic lines overexpressing *GmCBS21* compared with the control (Figure 6C, panels b–e N−).

During plant growth, sugar and nitrogen metabolism may regulate and control each other. In this study, more soluble sugar accumulated in NT and TL in LN treatment than in HN treatment, and the soluble sugar content of TL plants was slightly lower than that of the NT plants in the LN treatment (Figure 6C, panel f). No obvious differences were observed in the soluble sugar concentrations of NT and TL plants in HN treatment (Figure 6C, panel f).

### 2.7. Effects of GmCBS21 Expression on Amino Acid Composition

**Figure 7:**
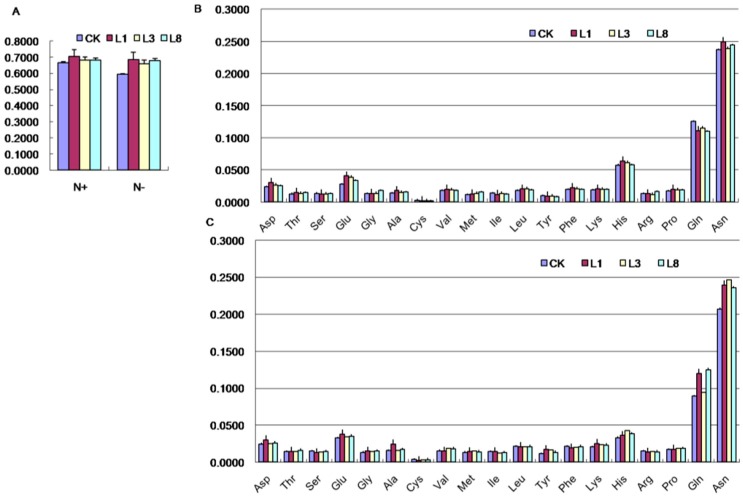
Concentrations of amino acids in transgenic and wild-type plants: (**A**) Concentrations of total amino acids; (**B**) Amino acid analysis at the normal N level; (**C**) Amino acid analysis at the low N level. L1, L3 and L8 were transgenic lines. CK was wild-type control line. *Arabidopsis thaliana* seedlings were treated with normal nitrogen (N+) and low nitrogen (N−). Data represent the mean value ± S.E. of three replicates.

To evaluate the effects of *GmCBS21* expression on nitrogen assimilation, the free amino acids in NT and TL plants were analyzed. The total free amino acid concentration of TL plants was higher than that of NT (Figure 7A). The concentrations of Asp, Glu, Gly, Ala, Met, Leu, Phe, Lys, His, Pro, and Asn were higher in TL than NT, and the concentrations of Ser, Cys, Tyr and Gln were lower in TL than NT under HN treatment (Figure 7B). Under LN stress, the concentrations of Asp, Thr, Glu, Gly, Ala, Met, Tyr, Lys, His, Pro, Gln, and Asn were higher in TL than NT, and the concentrations of Ser, Cys, and Ile were lower in TL than NT (Figure 7C). Glutamine and glutamate are good markers for nitrogen utilization. Increases in both Asn and Gln concentrations in the phloem sap during senescence can cause amino acid accumulation, and these residues are known to play a key role in rendering N available for remobilization from senescing leaves. Although increased amounts of several other amino acids were observed, the increases in Glu, Gln, and Asn were remarkable.

## 3. Discussion

The cystathionine-synthase (CBS) domain-containing proteins (CDCPs) consist of a large superfamily of evolutionarily conserved proteins, and these are present in all life science fields. Most of the CDCPs have been studied thus far in humans or other animals [11]. In plants, CDCPs have been reported infrequently, and hence their occurrence and possible function still need to be explored.

In recent years, some CDCPs such as IMPDH [15,17], AMPK [18] and ClC [19,20,21] have been studied in plants. However, *GmCBS21* has not been studied. At present, CDCPs with known functions that have been studied in soybean include IMPDH [22], ClC [23,24], and AMP-activated protein kinase. In order to determine the relationship between GmCBS21 and other CDCPs, a phylogenetic tree of the soybean CDCPs was constructed (Figure 2A). Our candidate protein, which is named GmCBS21, has low homology to other known proteins. The protein belongs to an independent unknown function branch. Therefore, exploring the function of *GmCBS21* is meaningful.

In this paper, to validate one of the CDCPs of unknown function, *GmCBS21*, a gene encoding a protein with a cystathionine-synthase (CBS) domain, was cloned from both 84–70 and Pohuang soybean because this gene was found to be differentially regulated (higher expression in Pohuang) among the contrasting genotypes in our previous study [4]. However, based on the sequencing result, no difference was found in the CDS or peptide sequence for the *GmCBS21* alleles between 84–70 and Pohuang (data not shown). These results indicate that higher expression levels of this gene in Pohuang might play a role in its low nitrogen tolerance.

CDCPs may play an important role in the development and stress response/tolerance of plants with respect to salinity, heavy metals, and oxidative stress [10]. However, the role of CDCPs in low nitrogen stress has not been investigated. In this study, the first description of an improvement in NUE under a nitrogen-deficient condition is the overexpression of *GmCBS21* in *Arabidopsis*. Three independent T2 homozygous lines were selected for further molecular and physiological analysis. When growing *Arabidopsis* plants for nine days under low nitrogen conditions, the transgenic lines with elevated *GmCBS21* expression had an improved phenotype compared with the control plants (Figure 6A). This included an increase in the fresh weight as well as the nitrogen concentration (Figure 6C).

**Figure 8:**
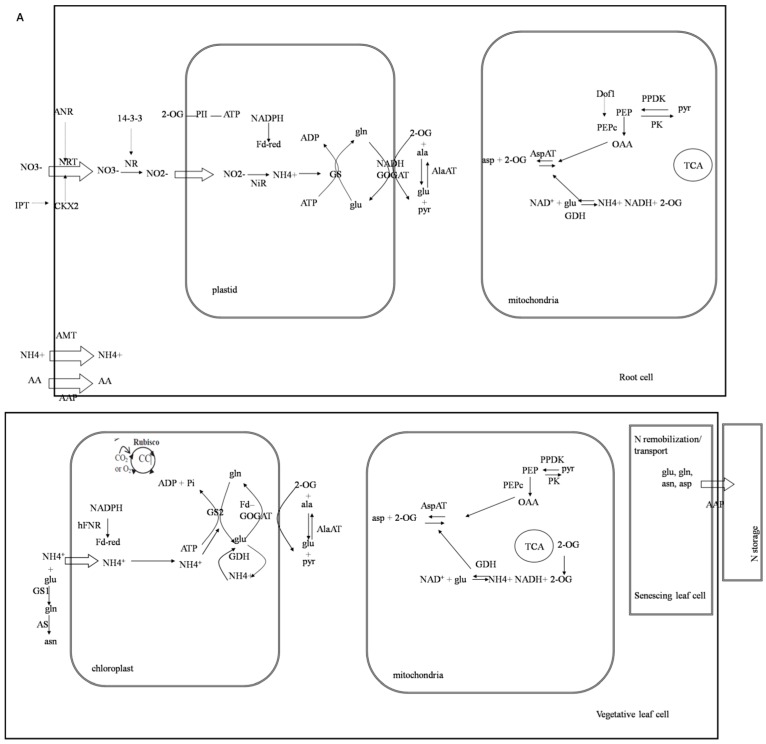

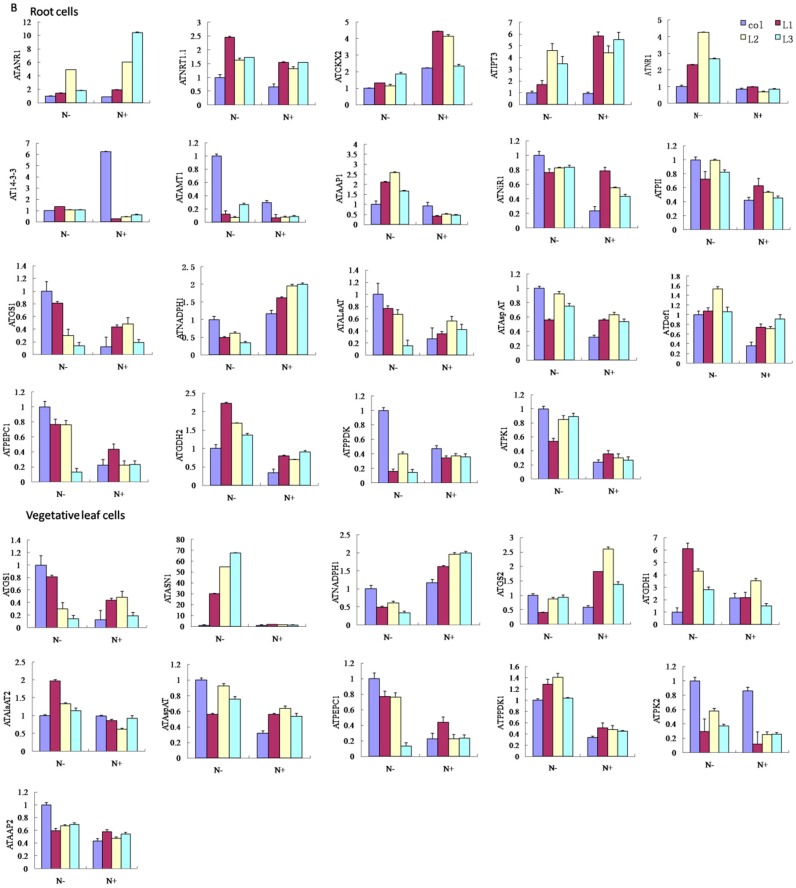
(**A**) The main processes of nitrogen utilization in plants; Dashed arrows represent transcript regulation, hollow arrows represent transport across membranes and black solid arrows represent an enzymatic reaction (**B**) The expression of genes involved in nitrogen uptake, assimilation, and remobilization of transgenic plants. L1, L3 and L8 were transgenic lines. Col was wild-type control line. AA, amino acids; AAT, amino acid transporter; AMT, ammonium transporter; NRT, nitrate transporter; 2-OG, 2-oxoglutarate; PK, pyruvate kinase; GS, glutamine synthetase; NR, nitrate reductase; AspAT, aspartate aminotransferase; PEPc, phosphoenolpyruvate carboxylase; GDH, glutamate dehydrogenase; AlaAT, alanine aminotransferase; IPT, isopentenyl transferase; CKX, cytokinin oxidase/dehydrogenase. L1, L3 and L8 were transgenic lines. Col was wild-type control line. Arabidopsis thaliana seedlings were treated with normal nitrogen (N+) and low nitrogen (N−). Data represent the mean value ± S.E. of three replicates.

Based on this correlative evidence between transcript accumulation and low nitrogen tolerance, we proposed that *GmCBS21* might play an important role in low nitrogen tolerance in plants. However, the mechanisms of low nitrogen tolerance conferred by *GmCBS21* overexpression plants remain unclear. We investigated the expression of several known genes involved in several main processes of nitrogen utilization in transgenic plants. The full name and the TAIR (The Arabidopsis Information Resource) coding number of these genes are shown in Table S6. The results are shown in Figure 8. The main frame of Figure 8 was based on previous work [25].

The main N forms absorbed by plants are water-soluble nitrate (NO_3_^−^), ammonium (NH_4_^+^), and to a lesser extent, proteins, peptides or amino acids [2]. The N uptake process requires the involvement of transporters. Dual-affinity nitrate transporter *NRT1.1* is a transceptor that senses the external nitrate concentration and activates *ANR1* (a MADS-box gene), which mediates the nitrate-signaling pathway to regulate nitrate-stimulated lateral root development [26,27]. The expression levels of *NRT1.1* and *ANR1* were increased in the *GmCBS21* transgenic plants compared with the wild type under conditions of normal (sufficient N) and low nitrogen (deficient N) (Figure 8). Cytokinins regulate NRT expression and act as an N satiety signal to decrease nitrate uptake by roots [28]. Nitrate-inducible *IPT3* (adenosine phosphate iso-pentenyl-transferase 3) is a key determinant of nitrate-dependent cytokinin biosynthesis [29]. The expression levels of *CKX2* and IPT3 were increased in the *GmCBS21* transgenic plants compared with the wild type under conditions of normal and low nitrogen. Ammonium uptake is facilitated by AMT transporters. The expression levels of *AMT1* were lower in the *GmCBS21* transgenic plants compared with the wild type under two nitrogen conditions (Figure 8). Plant roots can absorb small amounts of amino acids from the soil. Amino acid transporters play a role in this process. The expression levels of *AAT1* were increased in the *GmCBS21* transgenic plants compared with the wild type under conditions of low nitrogen, and were reduced under conditions of sufficient N.

Nitrogen assimilation occurs after nitrogen has been taken up by higher plants. NO_3_^−^ is reduced to NO_2_^−^ by nitrate reductase (NR). Under the low nitrogen condition, the expression level of NR was increased in the GmCBS21 transgenic plants compared with the wild type, but the expression level was similar to the wild type under the normal nitrogen condition. NO_2_^−^ is further reduced to ammonium (NH_4_^+^) by nitrite reductase (NiR). Under the low nitrogen condition, the expression levels of NiR in the *GmCBS21* transgenic plants were similar to the wild type, but were increased under the normal nitrogen condition. NH_4_^+^ is further assimilated into amino acids via the glutamine synthetase (GS)/glutamine-2-oxoglutarate aminotransferase (GOGAT) cycle. The expression level of GS1 and GS2 were lower under low nitrogen but higher under normal nitrogen. Another enzyme involved in NH_4_^+^ assimilation is glutamate dehydrogenase (GDH). The levels of GDH transcripts were much higher in the L1, L3, and L8 lines than in the wild type under both nutrition regimes. Asparagine synthetase (AS) catalyses the ATP-dependent transfer of the amido group of glutamine to a molecule of aspartate to generate glutamate and asparagine [30]. The expression levels of *ASN1* were significantly higher in the L1, L3, and L8 lines than in the wild type under the low nitrogen condition, but the expression levels showed no obvious change under the normal nitrogen condition. Two aminotransferase enzymes, aspartate aminotransferase (AspAT) and alanine aminotransferase (AlaAT), are important in amino acid biosynthesis. The expression levels of *AspAT* were much higher in the L1, L3, and L8 lines than in the wild type under the normal nitrogen condition, but the expression level was similar to the wild type under the low nitrogen condition. Under the normal nitrogen condition, the expression level of AlaAT in the *GmCBS21* transgenic plants was similar to that of the wild type. Under the low nitrogen condition, the expression level of *AlaAT* was lower in root cells and higher in vegetative leaf cells. PEPc is a key component in photosynthesis and is involved in the production of the keto-acid 2-oxoglutarate [31]. *Dof1* is involved in the activation of PEPc [32]. Under the normal nitrogen condition, the expression level of PEPc in the *GmCBS21* transgenic plants was similar to that of the wild type. Under the low nitrogen condition, the expression level of PEPc was lower. The expression level of *Dof1* was higher in the L1, L3, and L8 lines than in the wild type under both nutrition regimes. PII is an N-sensing and regulatory protein [33]. The expression level of PII in the *GmCBS21* transgenic plants was similar to that of the wild type. Pyruvate orthophosphate dikinase (PPDK) is involved in a pathway that generates the transport of the amino acid glutamine [34]. Pyruvate kinase (PK) catalyzes the transfer of a phosphate group from phosphoenolpyruvate (PEP) to ADP, yielding one molecule of pyruvate and one molecule of ATP. In root cells, the expression level of PPDK was significantly reduced in the L1, L3, and L8 lines relative to the wild type under the low nitrogen condition and the normal nitrogen condition. The expression level of PK was significantly increased in the L1, L3, and L8 lines relative to the wild type under the normal nitrogen condition. In vegetative leaf cells, the expression level of PK was significantly reduced in the L1, L3, and L8 lines relative to the wild type under the low nitrogen condition and the normal nitrogen condition. PPDK exhibited the opposite response. Amino acid permease (AAP1) is an integral membrane protein that catalyzes H^+^-coupled amino acid uptake [35]. The expression level of *AAP1* was significantly reduced in the L1, L3, and L8 lines relative to the wild type under the low nitrogen condition, but was similar in the wild-type and the L1, L3, and L8 seedlings under the normal nitrogen condition.

Under the low nitrogen condition, all transgenic plants produced high levels of mRNA for AlaAT, GDH, and AS. AlaAT catalyzes the reversible transfer of an amino group from glutamate to pyruvate to form 2-oxoglutarate and alanine. The regulation of *AlaAT* in several plant species has been studied in response to low oxygen stress light and nitrogen application, and it is involved in both carbon and nitrogen metabolism in plants [36]. Some studies discovered that overexpression of barley *AlaAT* in *Brassica napus* resulted in increased yield and biomass under N-limiting conditions compared with control plants [37]. Overexpression of AlaAT in transgenic rice plants with a tissue-specific promoter (OsAnt1) significantly increased biomass and grain yield [38]. Amino acids are catabolized by both glutamate GDH and transaminases. Ammonium is reassimilated by GS and, through the action of AS, is stored in asparagine (Asn). It has been reported that *Arabidopsis* plants deficient in GDH have shown decreased growth on reduced levels of C [39]. As previously reported, overexpression of AS with a CaMV35S promoter could enhance NUE in *Arabidopsis* plants; the transgenic plants had higher tolerance to low nitrogen when grown on plates and in the glasshouse [30]. Based on the above analyses, we speculate that overexpression of *GmCBS21* in transgenic plants activated the expression of *AlaAT*, *GDH*, and *AS* under low nitrogen conditions, thus affecting the low nitrogen tolerance of plants.

## 4. Materials and Methods

### 4.1. Database Searches for the Identification of CBS Domain-Containing Proteins Members in Soybean

Sequences of *Arabidopsis* and *Oryza sativa* CBS domain containing proteins (CDCPs) were retrieved from the *Arabidopsis* genome database, AIR 9.0 [40], the Rice Genome Annotation Project [41], and used as queries in BLAST searches against the soybean genome database of the Phytozome database) [10]. Sequences were selected for further analysis if the E value was below 1.0. A keyword search was conducted at the Phytozome (v9.0) database [42] for putative soybean CDCPs by searching ontologies with the term (PF00571) of CBS domain. If more than one transcript existed, the primary transcript was selected as representative. Then, we verified these sequences using the Pfam database [43] and SMART program [44]. Soybean-expressed sequence tag (EST) sequences were searched by blastn program in the Gene Indices at DFCI [45] using the transcript sequences of the identified putative soybean CBS genes as queries.

### 4.2. Phylogenetic, Motif Recognition, Gene Structure and Chromosomal Location Analyses

The multiple alignment analysis was performed with ClustalX 1.83 software [46]. Phylogenetic trees were generated by the neighbor-joining (NJ) method and bootstrap analysis (1000 replicates), and phylogenetic analysis was performed using MEGA5 software [47]. The conserved motifs were analyzed using MEME version 4.9.1 [48]. The exon/intron structures of CBS genes were determined by comparing the coding sequences and corresponding genomic sequences in the Gene Structure Display Server (GSDS) [49]. The chromosomal location of soybean CBS genes was generated using Chromosome Visualization Tool (CViT) at the Legume Information System [50].

### 4.3. Plant Materials and Growth Condition

Soybean seeds were germinated and grown hydroponically in half-strength modified Hoagland solution. The seedlings were grown for 10 days until the first trifoliate leaves fully developed, and then were grown with 10% of the normal N concentration. Leaves, stems, and roots were harvested separately after 0, 0.5, 2, 6, 9, and 12 h and 12 days of this treatment. Glycine max plants were used in this study. Pohuang and 84–70 cultivars were kept in our laboratory. T3 transgenic *Arabidopsis* and wild-type *Arabidopsis* plants were germinated and grown on MS media. After seven days, the seedlings were divided into two groups. One group of seedlings was transferred to low nitrogen (0.4 mM NO_3_^−^ and 0.2 mM NH_4_^+^, other ingredients refer to MS basic culture medium) MS media. For evaluation of soil-grown plants, another group of seedlings was transferred to pots containing vermiculite and Hoagland’s nutrient solution: (2 mM Ca(NO_3_)_2_·4H_2_O, 2.5 mM KNO_3_, 0.5 mM NH_4_NO_3_, 0.5 mM KH_2_PO_4_, 1 mM MgSO_4_·7H_2_O, 0.05 mM Fe-EDTA, 0.005 mM KI, 0.1 mM H_3_BO_3_, 0.1 mM MnSO_4_·H_2_O, 0.03 mM ZnSO_4_·7H_2_O, 0.0001 mM CuSO_4_·5H_2_O, 0.001 mM Na_2_MO_4_·2H_2_O, 0.0001 mM CoCl_2_·6H_2_O) to analyze the basal phenotype under the sufficient nutrient condition. Before bolting, the seedlings were treated with a nutrient solution without nitrogen or sufficient nitrogen. Samples were collected regularly to determine certain physiological indices.

### 4.4. RNA Extraction and Gene Expression Assays

Total RNA was extracted using TriZol reagent (Invitrogen, Carlsbad, CA, USA) and treated with DNaseI (Invitrogen) to avoid genomic DNA contamination. The first-strand cDNAs were synthesized using Superscript II reverse transcriptase (Invitrogen). The gene expression was analyzed by quantitative RT-PCR (qRT-PCR), which was performed with a SYBRR Premix ExTaq™ II kit (TaKaRa, Dalian, China) on an IQ™ 5 and MyiQ™ Real-Time PCR Detection Systems (Bio-Rad, Hercules, CA, USA). Table S6 lists the gene-specific and actin primers. The relative expression level is calculated using 2^−ΔΔ*C*t^ method.

### 4.5. Prokaryotic Expression and Cystathionine β-Synthase Activity Assay

Based on the sequencing results, the codons of the *GmCBS21* gene were optimized and the signal peptides were removed. The pCZN1-GmCBS21 plasmid was transduced into BL21 (DE3) competent cells for expression. The purified proteins were analyzed using SDS-PAGE. The cystathionine β-synthase activity of the purified protein was determined using the ninhydrin coloration method [51]. The absorbance at 454 nm was read against water and an enzyme-free blank was subtracted. The amount of cystathionine formed was determined relative to a standard, without enzyme, but containing 1 μmol of l-cystathionine.

### 4.6. Generation of GmCBS21 Transgenic Plants

The 1464 bp *GmCBS21* CDS was PCR amplified from the soybean genotype Pohuang. The cDNA fragment was ligated into the pCXSN vector driven by the CaMV 35S promoter. The hptII gene was used as a selection marker. All of the procedures of the experimental method followed standard molecular techniques. The plant expression vector pCXSN-GmCBS21 was transformed into *Arabidopsis* (Columbia) by the floral dip method [52] using the *Agrobacterium tumefaciens* strain GV3101. Seeds were screened using 100 mg·L^−1^ hygromycin. Hygromycin-resistant seedlings were self-pollinated to produce T2 generation seeds. The seedlings of each generation were tested using PCR and RT-PCR to confirm the transgenic status.

### 4.7. Quantification of Nitrogen, Soluble Sugar, Amino Acids

After 14 days of N-limited treatment, plants were collected and rapidly frozen in liquid nitrogen and then stored at −80 °C until analysis. Total nitrogen was determined using the Kjeldahl method [53]. Ammonium nitrogen and non-protein nitrogen were determined using colorimetric assays. Nitrate nitrogen was determined using an ultraviolet spectrophotometer. Soluble sugar contents were determined as described by Geiger [54]. For amino acid analysis, whole plants were ground in liquid nitrogen. The samples were hydrolyzed with HCL of 6 mol/L and analyzed using an automatic amino acid analyzer. Asparagine and glutamine were determined using HPLC.

## 5. Conclusions

In conclusion, we cloned a function-unknown gene (*GmCBS21*) from soybean, which had a CBS domain. Our research shows that *GmCBS21* does not belong to cystathionine β-synthase, and is different from other genes of known functions that contain a CBS domain. The *GmCBS21* protein is localized to the plasma membrane. Most importantly, overexpression of *GmCBS21* increased the tolerance of *Arabidopsis* under the low nitrogen condition, and it may play an important role in the nitrogen utilization efficiency of soybean. Although the precise molecular basis of improved tolerance to low nitrogen stress of *GmCBS21*-overexpressing transgenic *Arabidopsis* plants has not been completely resolved in this report, further characterization of the *GmCBS21* protein at the biochemical and molecular levels will provide insight into the exact nature and function of this protein in tolerance to low nitrogen stress.

## Supplementary Materials

Supplementary materials can be found at http://www.mdpi.com/1422-0067/17/5/620/s1.
