# Supplementary material for: Identification and Comparative Analysis of CBS Domain-Containing Proteins in Soybean (Glycine max) and the Primary Function of GmCBS21 in Enhanced Tolerance to Low Nitrogen Stress
Source: Int J Mol Sci. 2016 Apr 26;17(5):620. doi: 10.3390/ijms17050620 (PMC4881446; doi:10.3390/ijms17050620)
Supplement: Supplementary file 1 [file ijms-17-00620-s001.zip › ijms-123725-Supplementary Materials/ijms-123725-suppl..pdf]

Supplementary Materials: Identification and Comparative Analysis of CBS Domain-Containing Proteins in Soybean (*Glycine max*) and the Primary Function of *GmCBS21* in Enhanced Tolerance to Low Nitrogen Stress

Qingnan Hao, Weijuan Shang, Chanjuan Zhang, Haifeng Chen, Limiao Chen, Songli Yuan, Shuilian Chen, Xiaojuan Zhang and Xinan Zhou

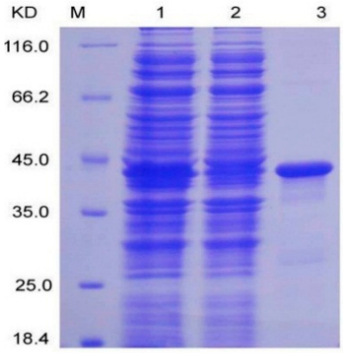

Figure S1. SDS-PAGE of pCZN1-GmCBS21-His fusion protein purification. M. Protein Marker; 1.Un-purified; 2. Flow through; 3 Elution.

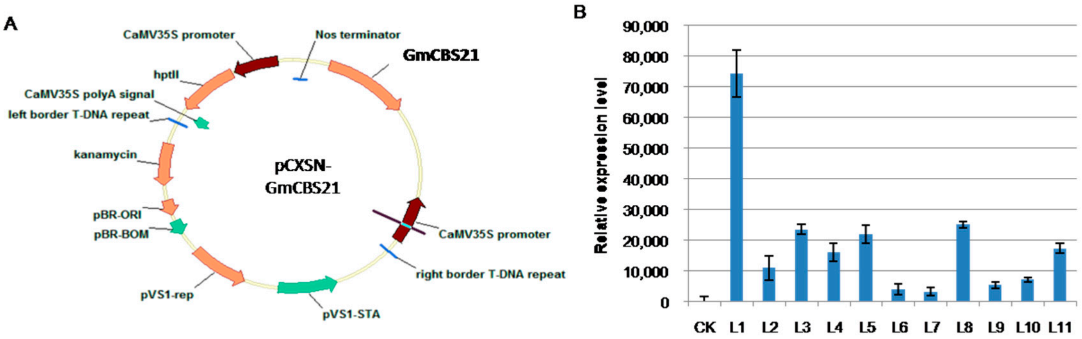

Figure S2. Generation and RT-PCR testing of GmCBS21-OXP lines. (**A**) GmCBS21 overexpression plasmid profile; (**B**) The relative expression level of GmCBS21-OXP lines.

Table S5. The calculation method of cystathionine activities.

Serial Number	S	B	A	Cystathionine (U) [(S − B)/A] × 2.2
1	2.112	1.937	0.124	
2	1.948	1.970	0.112	
3	1.916	1.979	0.125	[(S − B)/A] × 2.2 = [(2.010 − 1.960)/0.119] × 2.2 = 0.924
4	1.952	1.917	0.120	
5	2.123	1.995	0.113	
average	2.010	1.960	0.119	

S: assay group absorbance; value: control group absorbance; value A: absorbance value of 1 μmol cystathionine.

Table S6. The information of genes involved in nitrogen uptake, assimilation, and remobilization of transgenic plants.

Abbreviated Name	Full Name	TAIR Coding Number
ATANR1	MADS-BOX TRANSCRIPTION FACTOR ANR1	AT2G14210
AtNRT1.1	nitrate transporter 1.1	AT1G12110
AtCKX2	cytokinin oxidase 2	AT2G19500
AtIPT3	isopentenyltransferase 3	AT3G63110
AtNR1	nitrate reductase 1	AT1G77760
At14-3-3	14-3-3 family protein	AT1G22290
ATAMT1	ammonium transporter 1	AT4G13510
AtAAP1	amino acid permease 1	AT1G58360
AtNiR1	nitrite reductase 1	AT2G15620
AtPII	Nitrogen regulatory PII-like	AT2G33740
AtGS1	glutamate synthase 1	AT5G04140
AtNADPH1	NADH-dependent glutamate synthase 1	AT5G53460
AtAlaAT	alanine aminotransferas	AT1G17290
AtAspAT1	aspartate aminotransferase 1	AT2G30970
AtDof1	DOF zinc finger protein 1	AT1G51700
AtPEPC1	phosphoenolpyruvate carboxykinase 1	AT4G37870
AtGDH2	glutamate dehydrogenase 2	AT5G07440
AtPPDK	pyruvate orthophosphate dikinase	AT4G15530
AtPK1	pyruvate kinase family protein 1	AT3G49160
AtASN1	glutamine-dependent asparagine synthase 1	AT3G47340.1
AtGS2	glutamine synthetase 2	AT5G35630
AtGDH1	glutamate dehydrogenase 1	AT5G18170
AtAlaAT2	alanine aminotransferase 2	AT1G72330
AtAspAT	aspartate aminotransferase	AT2G22250
AtPK2	Pyruvate kinase family protein 2	AT3G22960
AtAAP2	amino acid permease 2	AT5G09220
